# Exploring the Role of Dairy Products In Sleep Quality: From Population Studies to Mechanistic Evaluations

**DOI:** 10.1016/j.advnut.2023.01.004

**Published:** 2023-01-31

**Authors:** Marie-Pierre St-Onge, Faris M. Zuraikat, Mackenzie Neilson

**Affiliations:** 1 Center of Excellence in Sleep and Circadian Research, Columbia University Irving Medical Center, New York, NY, USA; 2 Division of General Medicine, Department of Medicine, Columbia University Irving Medical Center, New York, NY, USA; 3 Institute of Human Nutrition, Columbia University Irving Medical Center, New York, NY, USA

**Keywords:** sleep quality, dairy intake, fermented dairy, dairy proteins, magnesium, zinc

## Abstract

Poor sleep quality and insufficient sleep affect a large portion of the population. This is concerning given increasing evidence that poor sleep health is a behavioral risk factor for the development of cardiometabolic diseases. A healthy diet is associated with a plethora of favorable health outcomes, and emerging research now highlights diet as a potential determinant of sleep health that could be leveraged to improve sleep quality. Dairy products are notably rich in tryptophan (Trp), a key substrate for serotonin and melatonin production, which are instrumental for initiating and maintaining sleep. Furthermore, dairy products provide a range of micronutrients that serve as cofactors in the synthesis of melatonin from Trp, which could contribute to sleep-promoting effects. In this review, we evaluate population studies and clinical trials to examine a possible link between dairy consumption and sleep. Available epidemiologic studies illustrate positive associations between dairy intake and sleep outcomes. Moreover, some intervention studies support a causal effect of dairy intake on sleep. Given these data, we discuss potential mechanisms, invite additional clinical research on this topic, and provide insights on how limitations of current studies can be addressed in future trials.

## Introduction

Sleep and diet are 2 lifestyle factors shown to predict cardiometabolic outcomes [[1], [2], [3]]. Short sleep duration, defined as sleeping <7 h/night [4], and poor quality sleep, defined as expressing dissatisfaction with sleep, having trouble sleeping, taking medications to sleep, or having difficulty staying awake during the day [5], are conditions that have been associated with higher risk of obesity [6] and cardiovascular disease [7]. Although the effect of sleep duration on dietary outcomes is established [8], there is now increasing evidence that this sleep–diet relation is bidirectional [9], with dietary intakes potentially influencing sleep health as well [10]. For example, it is postulated that consuming a higher proportion of protein in the diet could enhance sleep quality in healthy adults [11]. This has biological plausibility because melatonin and serotonin, 2 sleep-promoting agents, are synthesized from the essential amino acid tryptophan (Trp). Initial empirical support for this hypothesis is derived from animal studies showing that a Trp-rich diet efficiently restores sleep quality after starvation-induced poor sleep [12]. These studies led an early narrative review to conclude that foods with high Trp availability could be most helpful in improving sleep [13]. Among the various dietary sources of Trp, dairy products, such as milk, cottage cheese, hard cheeses, and yogurt, are notably rich in this amino acid.

Indeed, casein and whey, the main dairy proteins, are good sources of Trp [14]. Dairy products also provide a range of micronutrients with potential functional benefits for sleep [15, 16] owing to their roles as enzymatic cofactors in the metabolism of Trp. Vitamin B6, for example, is found in many dairy products and is essential to the production of serotonin from tryptophan, whereas magnesium and zinc are cofactors involved the synthesis pathway of serotonin to melatonin [17]. Given this nutrient profile of dairy products, along with other established cardiometabolic benefits of dairy consumption [[18], [19], [20]], we sought to evaluate the literature examining the role of this food group in sleep quality of adults. This review aimed to report findings from observational studies to establish the association between dairy consumption and sleep and to evaluate the causality from intervention studies. Finally, potential mechanisms by which dairy products could affect sleep quality are examined, and future actions to advance the field are proposed. We performed thorough PubMed searches between March and June 2021 and between April and June 2022 using words “dairy,” “milk,” “yogurt,” “cheese,” “fermented milk,” and “sleep” and “sleep quality,” filtering on adult studies. Our searches were restricted to original publications of epidemiologic and intervention studies.

## Association Between Dairy Consumption and Sleep: Epidemiologic Findings

Evaluation of population-level data can aid in establishing a link between dairy consumption and sleep (Table 1) [[21], [22], [23], [24], [25]]. One of the earliest epidemiologic studies to evaluate a potential link between dairy and sleep was a cross-sectional study among US college students [21]. Dairy products were categorized as either healthy (milk, yogurt, and cottage cheese) or unhealthy (all other cheeses), and sleep quality was quantified using the Pittsburgh Sleep Quality Index (PSQI). Results showed that greater frequency of consuming healthy dairy foods was related to lower odds of having poor sleep quality, with no relationship observed between unhealthy dairy products and sleep quality. Cheeses tend to have a high saturated fat content [26], greater intake of which has been shown to predict less deep sleep [27]. These findings highlight the importance of distinguishing between types of dairy products when assessing their influence on sleep.

**TABLE 1:** Summary of epidemiologic studies evaluating the relations between dairy intake and sleep quality

First author, year	Study population	Study design	Dairy exposure	Sleep outcome	Relevant findings
Knowlden et al., 2015 [21]	270 US students aged 18 y or older, M and F	Cross-sectional	Self-reported servings of dairy product per day based on FFQ: 1. Healthy dairy (milk, cottage cheese, yogurt) 2. Unhealthy dairy (cheese, processed cheese)	Sleep quality: poor (PSQI scores ≥5) or good (PSQI scores <5)	1. Healthy dairy: higher frequency of consumption related to lower odds of poor sleep 2. No association of unhealthy dairy intake with sleep quality
Hepsomali and Groeger, 2021 [22]	500,000 UK adults (from UK Biobank), 40–69 y, M and F	Cross-sectional	Milk intake scores, determined from estimated total milk intake per day based on FFQ: low, low/medium, medium, medium/high, high	Composite sleep score (range: 0–5), with higher scores representing better sleep	Curvilinear relationship between milk intake score and sleep score
					Sleep quality was lowest for high and low milk intake scores
Yasuda et al., 2019 [23]	679 Japanese elite athletes mean age: 25 y (range not provided), M and F	Cross-sectional	Self-reported servings of dairy intake based on frequency of consuming: 1. Milk + other dairy 2. Milk 3. Other dairy All exposures grouped as follows: low (0–2 d/wk), moderate (3–5 d/wk), high (6–7 d/wk)	Sleep quality: good or normal/poor	1. Milk + other dairy: not associated with sleep 2. Milk: moderate and high frequency of consumption related to lower odds of poor sleep among women only 3. Other dairy: moderate and high frequency of consumption tended to relate to lower odds of poor sleep across all subjects and in men
Kitano et al., 2014 [24]	421 Japanese adults aged 65 y or older, M and F	Cross-sectional	Frequency and quantity of dairy consumed using self-reported questionnaire: 1. Milk 2. Yogurt 3. Cheese 4. Total dairy Each intake exposure grouped as none, low, and high Physical activity: engaging or not engaging	DIS from PSQI sleep onset latency: no-DIS (≤30 min) or DIS (>30 min)	1. a) High level of milk intake related to lower odds for DIS b) Engaging in PA + consuming milk related to lower odds for DIS 2. a) Yogurt intake was not associated with DIS 3. a) Cheese intake was not associated with DIS b) Engaging in PA + consuming cheese related to lower odds for DIS 4. a) Total dairy was not associated with DIS
van Egmond et al., 2019 [25]	970 Swedish older adults (from Uppsala Longitudinal Study of Adult Men), aged 70 y or older, M only	Cross-sectional	Amount consumed of milk and dairy products per day using self-report: low (<median intake) or high (≥median intake)	DIS: Yes or no	Low milk and dairy intake related to lower odds for DIS
				DMS: Yes or no	No association of milk and dairy intake with DMS

DIS, difficulty initiating sleep; DMS, difficulty maintaining sleep; F, female; FFQ, food frequency questionnaire; M, male; PSQI, Pittsburgh Sleep Quality Index.

Another cross-sectional study that used data from 500,000 middle-aged adults in the UK Biobank examined the associations between milk intake and sleep [22]. Milk intake was estimated from questions related to the type of milk consumed and intake of foods and beverages consumed with milk (e.g., breakfast cereal, tea, and coffee). The results showed a curvilinear relation between milk intake and healthy sleep score; worse sleep scores were observed in the lowest and highest quintiles of milk intake. It is unsurprising that lower milk intakes were linked with poorer sleep in this cohort because milk contains 18 essential nutrients, including different B vitamins of particular importance to the homeostatic and circadian processes of sleep [[28], [29], [30], [31]]. These nutrients can also be obtained from other sources; because nutrients were not evaluated in this study, this hypothesis cannot be verified. However, that poorer sleep scores were observed at the highest level of milk intake is less intuitive. The researchers postulated that inflammation caused by excess saturated fat and D-galactose content with high milk intake may intensify poor sleep quality among adults [22]. Unfortunately, authors did not present intake data for total milk and did not distinguish between low-fat and high-fat milk profiles. Although this result may suggest an upper limit to any beneficial effect of dairy on sleep, any hypothesis about this or a role of the fat content of milk in the observed results are speculative.

To increase generalizability of a dairy–sleep relation, it is important to evaluate these factors across different populations and lifestyle patterns. Addressing this need was a cross-sectional study examining the association between frequency of milk or dairy consumption and sleep in Japanese athletes [23]. Results showed that greater frequency of milk consumption was related to better subjective sleep quality in female, but not male, athletes. Possible sex differences may be attributable to Trp hydroxylase activity, a key enzyme in the pathway linking Trp to melatonin, for which higher enzymatic activity with greater serotonin synthesis from Trp has been observed in females than males in murine models [32]. Further investigation into sex differences in Trp hydroxylase activity in humans is needed to confirm the mechanism, and more studies assessing sex differences in the association between Trp-rich foods and sleep are needed. Findings of such work would have important clinical implications for recommendations related to dietary changes for sleep health.

It is also notable that the population studied by Yasuda et al. [23], described in the previous paragraph, comprised athletes. Physical activity can affect both diet and sleep [33] and could, therefore, modulate the association of dairy intake with sleep. Insight into this potential interplay between lifestyle behaviors was provided by a study evaluating the associations between milk intake, physical activity, and sleep in older adults [24]. Overall, higher milk intake was associated with less difficulty initiating sleep. Moreover, adults who had both higher leisure-time physical activity and higher consumption of milk and/or cheese had better sleep than those with low physical activity and low dairy intakes. Exercise has been shown to increase Trp availability to the brain in older men [34], which could influence melatonin synthesis and improve sleep initiation.

The above-described studies suggest that associations between dairy consumption and sleep could be influenced by participant characteristics or other lifestyle factors, such as physical activity. Unfortunately, there is a sparsity of data on these potential individual differences. One study conducted exclusively in males showed that low dairy or milk intake was associated with lower odds of experiencing difficulty initiating sleep [28], contrasting the findings of the study by Yasuda et al. [27]. However, when male participants with implausible energy intakes, which constituted >50% of the sample, were excluded from the analyses, the association was no longer significant. Thus, we cannot preclude a potential sex difference in the relation between dairy and sleep, with the possibility that the association is larger in, or exclusive to, females. Furthermore, there is a need to investigate whether associations of dairy with sleep differ across other demographic factors such as race or ethnicity given the substantial disparities in sleep health [35] and diet. Another limitation of the epidemiologic evidence may be incomplete control of socioeconomic status and overall diet quality to allow a clear evaluation of the relationship between dairy and sleep.

At present, there are limited data from observational studies evaluating a role of dairy in sleep quality; however, existing results tend to support an association between these factors. Despite some positive findings, there are notable limitations: *1*) food intake and sleep were assessed by self-report measures, which are prone to bias; *2*) studies were conducted among adults with healthy sleep; and *3*) epidemiologic studies lack ability to ascribe causality, or even directionality, to associations. In addition, the lack of correction for covariates that might affect sleep quality and diet, such as medication use, and attention to potential moderators, such as age and sex, are not clearly addressed in current cross-sectional studies. Some of these limitations can be resolved using existing data from large prospective cohort studies, such as the Multi-Ethnic Study of Atherosclerosis and The Hispanic Community Health Study, which incorporate detailed evaluation of participant characteristics and health history, longitudinal assessments of diet, and objective measures of sleep [[36], [37], [38]].

## The Effect of Milk-Based Mixtures on Sleep: Intervention Studies

Experimental variations in the amount or type of dairy consumed can be used to elucidate a causal pathway linking dairy with sleep outcomes (Table 2) [[39], [40], [41], [42], [43], [44], [45], [46]]. Work dating back to 1934 has examined the influence of a light meal of corn flakes and milk, compared with a “hard-to-digest” meal of undisclosed composition on sleep quality in 8 adults and 8 children [47]. All participants had fewer movements in their sleep after the cereal and milk supper compared with the hard-to-digest supper. In more recent times, seminal studies investigating the effect of dairy on sleep quality used Horlicks with milk as the test beverage [48, 49]. Horlicks milk is a malted beverage composed of milk and grain-based powder marketed as a bedtime snack to promote sleep in the 1960s and 1970s. In a study, 4 male participants were assessed twice under each of 3 conditions: no beverage, 350 mL of water, and 350 mL of warm milk with 5 teaspoons of Horlicks powder [48]. Beverages were consumed immediately before bedtime and participants had a 6-h sleep opportunity. Consumption of the milk-based drink led to a reduction in small movements throughout the night, quantified by lapse cinematography, whereas movements increased throughout the night in the control and water conditions. In a subsequent study, older and younger adults (8 male and 9 female participants) received either an inert capsule that they were told was developed based on a folk remedy for sleep promotion or a mixed cereal drink composed of Horlicks powder and warm milk immediately before bedtime [49]. After a 2-night habituation period, sleep quality was assessed using electroencephalography during each of 5 nights of either placebo or Horlicks milk, in a random order. Young adults tended to have fewer movements during the last 3 h of the 9-h sleep opportunity in the Horlicks condition compared with placebo. The authors report that younger female participants experienced a longer sleep duration, earlier sleep onset, and less wake after sleep onset in Horlicks milk condition compared with placebo. Older adults experienced longer mean total sleep time and less wake after sleep onset in the Horlicks condition compared with placebo. As such, consumption of Horlicks milk improved sleep quality across age groups, providing the first evidence of a causal effect on sleep of consuming a milk-based beverage before bed. Unfortunately, we cannot discern from these studies [48, 49] whether favorable effects on sleep quality observed were due to the milk, the Horlicks powder, or their combination. It is plausible that effects were driven by the protein-rich milk and its intake in conjunction with carbohydrates from the Horlicks powder, which can improve Trp availability for brain uptake. Carbohydrates increase the ratio of Trp to other large neutral amino acids (LNAA) in circulation [39], enhancing its uptake by the brain thereby potentially enhancing the synthesis of serotonin and melatonin.

**TABLE 2:** Summary of human clinical trials testing the effects of dairy products and dairy proteins on sleep quality

Author, year	Study population	Study design	Levels of independent variable	Sleep outcome	Key findings
Fakhr-Movahedi et al., 2018 [40]	68 Iranian patients with acute coronary syndrome, aged 35–85 y, M and F	Randomized, controlled, parallel arm	Experimental: Milk (150 mL)–honey (30 g) mixture, twice/d for 3 d	Sleep quality score measured using Richards-Campbell Sleep Questionnaire at baseline and day 3	Sleep quality increased in the experimental condition; no change in control (no statistical comparison of these changes)
			Control: Patients received routine care. No beverage provided		Sleep quality at day 3 was higher in the experimental group than in the control group
Schaafsma et al., 2021 [50]	70 Dutch healthy adults, aged 30–50 y, M and F	Double-blind, placebo-controlled, randomized crossover	Experimental: 19 g of dairy whey protein and GOS-based beverage enriched with Trp (see text for more detail)	Sleep quality measured using PSQI weekly	No differences in change in sleep quality observed in an intention-to-treat analysis
			Control: 12 g of taste-matched skimmed milk powder placebo beverage	Multiple sleep dimensions measured objectively over 5 d using headband tracker at baseline and end point	In modified per-protocol analysis, at day 14, sleep quality improved in the experimental compared with that in the control group
			Experimental and placebo beverages consumed daily for 3 wk		Objectively measured REM sleep improved in the experimental group compared with that in the control group
Özcan et al., 2019 [42]	68 Turkish menopausal women with sleep complaints, aged 45–65 y, F only	Randomized controlled, parallel arm	Experimental: 500 mL kefir beverage daily for 1 mo	Sleep complaints measured using WHIIRS at baseline and end point (lower scores represent better sleep)	Sleep improved (decrease in WHIIRS score) in the experimental condition; no change in control (no statistical comparison of these changes)
			Control: No beverage provided		End point sleep scores were lower (better sleep) in the experimental vs. control
Yamamura et al., 2009 [44]	29 Japanese older healthy adults, aged 60–81 y, M and F	Double-blind, placebo-controlled, randomized crossover	Experimental: 100 g fermented milk beverage with *Lactobacillus helveticus*	Multiple sleep dimensions measured using wrist actigraphy over 3 wk	Sleep efficiency increased and WASO decreased in the experimental condition; no change in control (no statistical comparison of these changes)
			Control: 100 g placebo milk matched for acidity	Sleep health risk measured using Sleep Health Risk Index score at end point	No between-condition differences in end point measures
			Experimental and control beverages consumed daily for daily for 3 wk		
Kinoshita et al., 2021 [52]	961 Japanese health care workers, aged 20–71 y, F only	Randomized, controlled, open label	Experimental: 112 mL fermented yogurt with *Lactobacillus delbrueckii* ssp. *bulgaricus OLL1073R-1* consumed daily for 16 wk	Sleep quality measured using PSQI at baseline and end point	Sleep quality improved (PSQI score decreased) in experimental vs. control
			Control: No yogurt		
Takada et al., 2017 [43]	94 Japanese healthy students, aged 30 y or younger, M and F	Double-blind, placebo-controlled, parallel arm	Experimental: 100 mL fermented milk with *Lactobacillus casei*	Sleep quality measured using Oguri-Shirakawa-Azumi Sleep Inventory	No between-group differences in changes in total sleep score
			Control: 100 mL placebo milk matched for acidity and flavor	Multiple sleep dimensions measured using single-channel EEG	Self-reported sleepiness decreased (higher scores represent less sleepiness) and sleep time increased in experimental vs. control
			Experimental and control beverages consumed daily for 11 wk (8 wk until stressful stimulus and 3 wk after)	Measures collected at baseline, wk 6, wk 8, wk 9, and wk 11	N3 stage sleep and δ power was decreased in control vs. experimental
Markus et al., 2005 [57]	28 Dutch healthy students: (14 with sleep complaints, 14 without), mean age: 22 y (range not provided), M and F	Double-blind, placebo-controlled, randomized crossover	Experimental: Milkshake with 20 g of Trp-enriched A-LAC	Sleepiness measured using Stanford Sleepiness Scale in the evening before and morning after the meal	Trp:LNAA was higher in experimental vs. control; observed in both poor and good habitual sleepers
			Control: Energy-matched placebo milkshake with 20 g sodium caseinate	Plasma Trp:LNAA levels after the meal	Sleepiness decreased in experimental vs control; observed in both good and poor habitual sleepers
			Beverages consumed with standard meal on 1 night		
Ong et al., 2017 [46]	10 Australian college students, mean age: 27 y (range not provided), M only	Double-blind, placebo-controlled, randomized crossover	Experimental: Milkshake with 20 g of Trp-enriched A-LAC	Multiple sleep dimensions measured using wrist actigraphy	Total sleep time was significantly higher in experimental vs. control
			Control: Energy-matched placebo milkshake with 20 g sodium caseinate		Sleep efficiency was significantly higher in experimental vs. control
			Beverages consumed with standard meal on 2 nights		

A-LAC, α-lactalbumin; DIS, difficulty initiating sleep; DMS, difficulty maintaining sleep; EEG, electroencephalogram; F, female; GOS, galacto-oligosaccaride; LNAA, large neutral amino acid; M, male; PSQI, Pittsburgh Sleep Quality Index; REM, rapid eye movement; Trp, tryptophan; WASO, wake after sleep onset; WHIIRS, Women’s Health Initiative Insomnia Rating Scale.

Even more recent studies have assessed changes in sleep in response to intake of other milk-based beverages. In a randomized controlled trial, 68 patients with acute coronary syndrome were provided either a milk–honey beverage twice daily for 3 days or no beverage [40]. Participants assigned to the milk–honey beverage reported better subjective sleep quality relative to the control group. Although the mechanism underlying this beneficial effect of a milk–honey mixture cannot be determined from the study, we speculate that honey, a source of carbohydrates, may have increased Trp availability for serotonin and melatonin synthesis.

Another recent study investigated the effects of a whey protein–galacto-oligosaccharide (GOS) product on sleep quality in healthy Dutch men and women reporting mild to moderate sleep disturbances [50]. In this crossover study, participants consumed 2 dairy-based beverages nightly over 3-week periods in a random order. The experimental condition included a GOS beverage fortified with Trp, tryptic casein hydrolysate, magnesium, zinc, niacin, vitamin B6, and vitamin D3; the control condition included a taste-matched milk placebo without any additives. Sleep was assessed by questionnaire and headband sleep tracker at the beginning and at the end of each period, with results demonstrating that rapid eye movement sleep was 10 min longer in GOS condition relative to control. Moreover, participants reported a better sleep quality after 2 weeks of the experimental condition relative to control, but this effect was limited to participants with a poor sleep quality at baseline and did not persist beyond week 2. Despite the need for replication in a larger sample, this study provides initial insight into the potential benefits of the sleep-enhancing nutrients incorporated into the whey protein–GOS product and builds ground to produce future studies to assess similar outcomes with enhanced methodology. For example, polysomnography should be administered at multiple timepoints to evaluate changes in sleep architecture over time in response to the experimental beverage. In addition, longer intervention periods, provision of a controlled diet, and inclusion of a nondairy control beverage would allow for a better characterization of the effect of the whey protein–GOS drink on sleep parameters. Finally, studies specifically in participants with self-reported poor sleep are needed.

## The Effect of Fermented Dairy Products on Sleep Quality

It is also plausible that dairy products could affect sleep by promoting favorable changes in the gut microbiome [51]. Fermented milk products, rich in prebiotics and probiotics, promote favorable health outcomes [41]. These products may confer additional, or differential, sleep benefits relative to nonfermented dairy products by influencing the gut microbiome.

Clinical trials have tested the influence of fermented dairy products on sleep. One such study measured changes in insomnia symptoms in postmenopausal women with long-term sleep complaints [42]. These women, aged 45–65 y, were randomly assigned to consume 250 mL of kefir, a fermented milk drink, twice daily for 30 days or no beverage. Mean ratings of insomnia symptoms decreased substantially from baseline (10.8 ± 4.4) to endpoint (3.5 ± 3.4) in the kefir group but remained stable in the control group (baseline: 9.4 ± 4.5; end point: 9.9 ± 5.0). Hence, the experimental group showed significantly lower self-reported insomnia scores at endpoint compared with the controls. Another trial assessed the effect of *Lactobacillus*-fermented milk on sleep in elderly adults [44]. In this randomized, double-blind, placebo-controlled crossover study, participants consumed 100 g/d of fermented milk or acidified milk for 3 weeks. Sleep efficiency and number of wake episodes, measured by actigraphy, improved from baseline with fermented milk consumption. By contrast, no changes in these sleep outcomes were observed in the acidified milk condition. Endpoint measures did not differ significantly between conditions owing to nonsignificant improvements in the acidified milk condition. It is possible that any milk consumption may improve sleep in this population, highlighting the need to include a nondairy control beverage in intervention studies.

Another randomized, double blind, placebo-controlled trial among medical students in Japan, tested whether *Lactobacillus casei* strain *Shirota* (LcS)-fermented milk decreased stress through improved sleep quality before and after a national standardized test compared with a placebo milk drink [43]. Objectively measured slow-wave sleep decreased in the placebo group as the examination approached with no impairment in this sleep parameter observed in the LcS group, indicating positive effects of LcS on sleep depth. In addition, the increase in sleep latency in the weeks leading to the examination observed in the placebo group was not seen in the LcS group and differences in sleep onset latency remained after examinations. Together, the results, although preliminary, suggest that consumption of fermented milk may protect against stress-induced declines in sleep quality and could improve sleep recovery after stressful events.

In addition to milk, yogurt fermented with *Lactobacillus delbrueckii* ssp. *bulgaricus* OLL1073R-1 has also been evaluated for potential sleep-promoting effects. In a study of 960 female healthcare workers, participants were randomly assigned to consume 112 mL of yogurt daily or to avoid all yogurt and fermented dairy products for 16 weeks; sleep was assessed using the PSQI [52]. Self-reported sleep quality improved among those assigned to yogurt relative to the control group. The yogurt group also improved in perceived general health and vitality, scores for which were associated with improvements in sleep quality. Unfortunately, it is difficult to draw firm conclusions on the direct role of the fermented yogurt in improving sleep outcomes in this study because the researchers did not evaluate habitual probiotic intake at baseline, nor did they measure changes in gut microbiota to confirm the mechanism. Although the absence of baseline data is a limitation, it seems more likely that yogurt intakes were lower at study onset and increased in the experimental group rather than high at study onset and reduced in the control group. Thus, findings align with results of other studies investigating how consumption of fermented dairy products, such as yogurt, may improve sleep quality.

**FIGURE 1:**
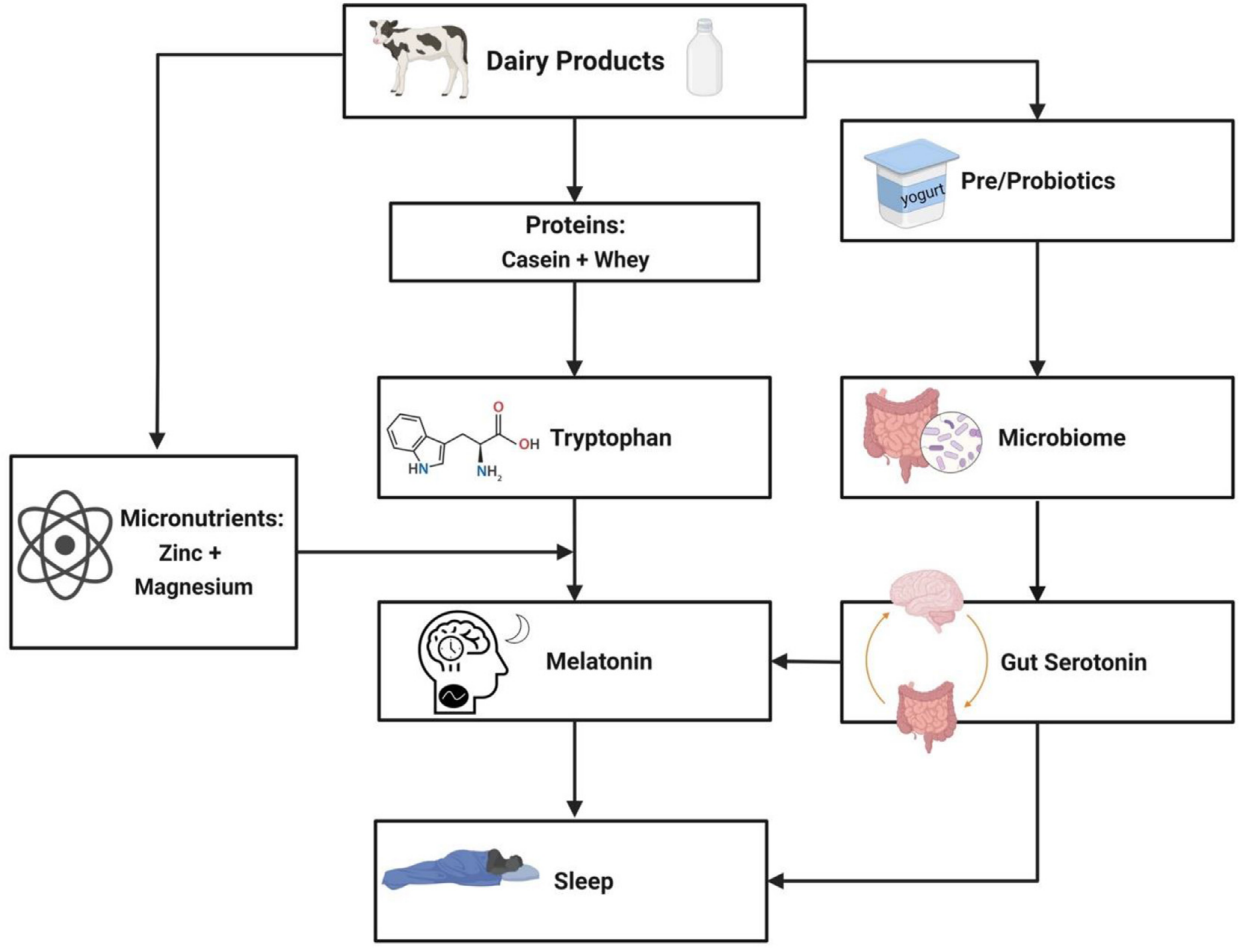
Consumption of dairy products could promote sleep quality by increasing endogenous melatonin production. First, the primary protein sources in dairy, casein and whey, are rich in tryptophan (Trp). Given that Trp is a precursor for the sleep-promoting hormone melatonin (through serotonin), dairy proteins may favorably affect sleep quality by increasing Trp intake. Augmenting the potential role of dairy intake in sleep is its content of micronutrients involved in enzymatic conversions of Trp to serotonin and melatonin. Dairy products are good sources of zinc and magnesium, which serve as cofactors in the production of melatonin from serotonin. Finally, fermented dairy products may exert beneficial effects on sleep by altering gut microbial composition that favor production of serotonin, which can then be converted to melatonin.

Fermented dairy products could affect sleep quality by modulating the gut microbiota composition (Figure 1). A systematic review noted that dairy products increase the abundance of beneficial gut bacteria, such as *Lactobacillus* and *Bifidobacterium*, and can decrease the abundance of detrimental strains, such as *Bacteroides fragilis* [53]. Favorable health outcomes associated with the consumption of dairy and fermented dairy products could be modulated, in part, by changes in host bacteria [41]. Regarding mechanisms of action, Takada et al. [43] have suggested that modifications in the gut microbiota may improve sleep through reduced activation in gut–brain pathways promoting arousal. However, because much of the literature stems from rodent models or observational studies, we cannot draw firm conclusions. Human clinical trials should be developed to test a potential modulating effect of the gut microbiome on the effect of dairy on sleep. These studies should also be designed to evaluate potential individual differences because microbiome content can vary across a variety of host-related factors, such as age and dietary nutrient density [54]. Characterizing individual responses of the gut microbiome to dairy intake could be used to develop personalized dietary strategies to improve sleep, aligning with the growing priority to enhance precision nutrition approaches to health [55].

## The Effects of Dairy Proteins on Sleep

The biochemistry of endogenous melatonin production is such that dairy products could affect sleep through their nutrient profiles, which would promote melatonin synthesis by increasing circulating Trp (Figure 1). Indeed, dairy proteins, such as casein, whey, and α-lactalbumin (A-LAC), are complete proteins and thus provide Trp, the amino acid precursor of melatonin [56]. Dairy proteins like A-LAC, have been shown to increase the proportion of plasma Trp when enriched in the diet [45]. Hence, natural or experimental variations to levels of dairy proteins in foods and beverages is of interest when investigating sleep quality.

### Dairy proteins and sleep: A-LAC

The earliest insight into the role of dairy proteins in determining sleep quality came from a study that varied the types and quantities of dairy protein in a rodent model [12]. Rats were deprived of food for 4 days, which reduced their sleep duration and increased time to fall asleep. Then, rats were refed a diet that included either 30% whole milk protein, 30% A-LAC–enriched protein, or 14% whole milk protein for 6 days. Sleep quality recovered immediately in rats refed with the A-LAC–enriched diet; time spent in slow-wave sleep increased and length of wake episodes decreased compared with those at baseline. Refeeding rats with a 30% whole milk diet also progressively restored sleep over 5–6 days, an improvement that was generally better than that observed in rats refed with the 14% whole milk diet. These data suggest that higher intakes of whole milk protein and, more specifically, A-LAC protein from milk, leads to more rapid rebounds in sleep after periods of poor sleep. However, translation of these findings to humans is needed because results could provide insight into a feasible strategy to improve sleep quality in poor sleepers.

The potential clinical significance of a favorable effect on sleep of supplementing the diet with dairy protein and consequent need for translational work was recognized, and clinical trials have been developed to evaluate whether these effects extend to humans [46, 57]. In a trial, 14 participants with poor sleep and 14 without poor sleep consumed a shake with 20 g A-LAC and high Trp concentration (4.8 g/100 g) protein or a placebo shake containing 20 g sodium caseinate with the evening meal and 1 h later [57]. Levels of Trp:LNAA and sleepiness were assessed 2 h after the second shake, and sleepiness was measured again the following morning of each experimental condition. Levels of Trp:LNAA were elevated when participants consumed the A-LAC compared with the placebo shake, an effect observed across poor and nonpoor habitual sleepers. Moreover, consumption of A-LAC shakes led to reductions in sleepiness relative to control among all participants and improved reaction time among those with a poor baseline sleep. The authors speculated that consuming Trp in the evening improved performance in the morning owing to better nighttime sleep from greater Trp availability to the brain [57].

Further support for a sleep-promoting role of A-LAC in humans was provided from a double-blind randomized crossover trial among 10 young male participants with healthy habitual sleep [46]. Participants received a standardized meal, followed by an evening shake containing 20 g A-LAC or 20 g sodium caseinate protein on 2 nights. Sleep duration and efficiency were significantly higher after consumption of the A-LAC shake compared with after the control shake consumption. These effects are likely explained by improved sleep initiation because time to fall asleep was reduced in the A-LAC condition relative to the control [46]. Although not measured in this study, findings may be indicative of increased melatonin synthesis or secretion from the pineal gland, downstream of heightened brain Trp uptake in response to the high Trp:LNAA of A-LAC. It is interesting that these improvements in sleep were observed among individuals with sufficient habitual sleep duration. While it might be expected that effects would be larger among those with habitually poor sleep or insomnia symptoms given their difficulty with initiating sleep, empirical evidence of this is needed.

### Dairy proteins and sleep: α-s1 casein hydrolysate

Other studies have evaluated the effects of α-s1 casein tryptic hydrolysate (CTH), another dairy protein, on sleep disturbances in rodents [58, 59] and humans [60]. In rats, consumption of CTH ameliorates chronic stress-induced sleep disturbances as evidenced by maintained slow-wave sleep and increased paradoxical sleep duration [58]. Building from this, researchers found that rodents injected with CTH had fewer sleep/wake cycles and increased total sleep time compared with saline-treated control mice [59]. The authors suggested that improvements in sleep were likely due to increase in GABA_A_ receptor B1 subunit protein expression, a known target for insomnia treatment, and proposed dietary CTH supplementation as a potential treatment for sleep disorders.

Given promising data linking CTH with sleep in murine models, studies in humans followed. Among a community-based sample of adults with poor sleep quality but no diagnosed sleep disorders, researchers randomly assigned individuals to receive either placebo or CTH capsules (300 mg/d) for 4 weeks in a crossover design [60]. Although self-reported measures of sleep disturbances improved slightly over time with consumption of the CTH capsule, neither endpoint measures nor their changes over time differed between conditions. By contrast, actigraphy-derived sleep efficiency improved to a greater extent over 4 weeks in the CTH condition relative to control, whereas both wake after sleep onset and time to fall asleep tended to be reduced in the experimental compared with control conditions. Furthermore, data were more pronounced at the 4-week assessment time point than at the 2-week time point, probing the question of a potential continued improvement in sleep with further prolonged intake. Although results are not definitive, it is possible that supplementing the diet with CTH could favorably affect sleep quality, particularly if CTH consumption is sustained over a longer period. While the pathway linking CTH with sleep outcomes remains to be elucidated, there are at least 2 plausible mechanisms: *1*) Trp concentration of CTH increases melatonin production, and *2*) CTH increases GABA_A_ B1 receptor expression.

The hydrolysis of milk involves proteins that could also act on sleep quantity or quality. Most notable among these are CTH and α-casozepine, a derivative of CTH [61]. These peptides are found in cow’s milk and have been shown to reduce anxiety and insomnia through GABA receptor activity across different animal models [58, [62], [63], [64]]. In a study, the potential sleep-enhancing effects of CTH and α-casozepine peptides were explored in mice [65]. The researchers evaluated sleep latency and sleep duration following an oral ingestion of water (control), 1 mg/kg of a benzodiazepine (positive control), 50 mg/kg CTH, 100 mg/kg CTH, or 2 mg/kg α-casozepine and enumerating mice that fell asleep within 15 min after feeding across conditions. All treatments improved sleep duration, with the greatest increases observed in response to the larger dose of CTH. Further exploratory analysis revealed bioactive peptides of CTH that demonstrated particularly high affinity for binding with the GABA_A_ receptor, with some indication of differential magnitudes of sleep-enhancing effects across peptides.

Consumption of dairy proteins seems to improve sleep quality. However, uncertainty prevails because the mechanisms underlying the effects are not confirmed. Longer-term, tightly controlled studies are needed to firmly establish effects and to elucidate the mechanistic pathways. At present, the strongest body of evidence is for a sleep-promoting role of A-LAC, likely driven by increased Trp consumption.

## Micronutrients Common to Dairy and Involved in the Tryptophan-to-Melatonin Conversion Pathway: Do They Have a Role in Sleep?

Micronutrients, particularly magnesium and zinc, are essential to the production of melatonin from dietary Trp, acting as key cofactors to enzymatic reactions in this pathway [10]. Notably, dairy products are rich sources of these micronutrients [66]. Thus, investigation of the role of these micronutrients, ubiquitous in dairy products, in determining sleep outcomes could provide key insight into how increasing dairy intake might improve sleep.

### Magnesium and zinc in relation to sleep outcomes: epidemiologic studies

The relationships of magnesium and zinc with sleep have been explored in epidemiologic studies. Results of a systematic review concluded that greater intakes of magnesium and zinc are associated with greater time spent in deep sleep and earlier sleep timing, respectively [67]. In addition, data from observational studies show a positive relationship between zinc intake and sleep duration [68, 69]. A recent analysis using data from the National Health and Nutrition Examination Survey showed that participants with lower intakes of magnesium tended to have shorter sleep duration [70]. Similarly, in the Coronary Artery Risk Development in Young Adults study, those with the highest intakes of magnesium tended to report better sleep quality and showed lower odds of not achieving sufficient sleep duration of ≥7 h/night [71]. These observational data confirm a relationship between micronutrient cofactors of the Trp-melatonin pathway and sleep outcomes (Figure 1). However, these studies include the total intake of magnesium and/or zinc and not solely dairy contribution. Nevertheless, it is plausible that these nutrients contribute to the mechanism by which dairy products could improve sleep.

### Magnesium, zinc, and sleep: clinical trials

Epidemiologic findings of relations between micronutrients in dairy products and sleep have been extended through clinical trials. A double-blind randomized controlled trial in elderly adults with insomnia tested the effect of 500 mg/d of magnesium or placebo for 8 weeks on sleep [72]. Participants receiving magnesium supplements showed greater improvements in insomnia symptoms, including sleep onset latency and sleep efficiency, than those receiving placebo. The magnesium supplement group also recorded increased serum melatonin levels compared with the control group, suggesting that magnesium supplementation could enhance melatonin synthesis. However, average serum magnesium for participants in this study was below normal values at baseline. Magnesium deficiency has been proposed to play a role in age-related changes in sleep architecture in older adults [73]. It is possible that poor sleep is a consequence of magnesium deficiency and that restoring adequacy improves sleep. Whether similar findings would be observed in those with already adequate magnesium status remains to be determined.

Another dietary supplement study was performed in 22 elderly adults residing in a long-term care facility [74]. Participants received a placebo or a dietary supplement that comprised 5 mg of melatonin, 225 mg of magnesium, and 11.25 mg of zinc daily, 1 h before bedtime for 8 weeks. Participants in the dietary supplement group showed greater improvements in sleep quality, assessed using the PSQI, relative to the placebo group. Improvements in all 4 domains of the Leeds Sleep Evaluation Questionnaire (ease of falling asleep, quality of sleep, morning alertness, and ease of awakening) were observed as well. However, because the active supplement comprised of a medley of compounds, such as melatonin, the effects cannot be ascribed to any 1 component. Melatonin alone could be responsible for improvements in sleep [75]. A future study that provides these compounds separately and in various combinations could address this question.

## Discussion

Although available evidence points towards a beneficial role of dairy products in sleep quality, several limitations preclude the development of firm conclusions. First, few clinical intervention studies are available. Additional studies with larger samples are needed to confirm the causal effect of dairy products and their constituents on sleep quality and to elucidate the effect of dairy products in different participant groups such as male and female participants, younger and older adults, athletes and nonathletes, and, importantly, poor and good sleepers [e.g., those with insomnia symptoms [76] or poor quality sleep [5]]. Appropriateness of the control product also deserves attention. For example, a non-dairy food/beverage should be included in all studies so that comparisons between dairy products could be assessed among each other and relative to non-dairy products. Failure to observe effects on sleep when comparing differing dairy products may be because of intervention and control products both influencing sleep quality. Several modulating factors may influence the effect of dairy products such as inclusion of additional carbohydrates or presence of bacteria. Dairy products are complex food matrices that include multiple nutrients that could influence sleep. Hence, it may be difficult to disentangle the effects of dairy from other dietary components. This is a limitation of all epidemiologic nutrition studies that can only be overcome by tightly controlled feeding studies. Moreover, even in these trials, many nonnutritive, often unmeasurable components of the diet, such as phytonutrients or other polyphenols, may influence sleep in a myriad of ways. In this study, we have focused on the protein and micronutrient profiles but the contribution of those nutrients, and of non-nutrient components in dairy, should also be examined, as has been recommended for other health fields [77].

Future studies should strive to include both self-reported sleep measures, such as questionnaires of sleep quality and its components (e.g., satisfaction, daytime dysfunction, sleep disturbances) [5] and objective sleep measures, such as actigraphy or polysomnography, to fully capture the multidimensionality of sleep quality. It is important to reiterate that this work should be conducted among adults with sleep disorders because they would be the target population for whom dietary treatments would be developed and recommended.

It is also worth discussing whether dairy alternatives produce comparable effects on sleep as their dairy counterparts. Many of those replacement products are fortified with calcium, vitamin A, vitamin D, and, less frequently, with vitamins B12 and E and zinc [78], nutrients that may confer sleep benefits. However, there are presently no nutrient standards in place for those products, which also tend to include sweeteners and are low in protein. Standards have been proposed to enhance nutrient density of milk and dairy alternatives, but only 5% of currently available plant-based beverages, all of which were soy based, meet these standards [78]. Given that a potential role of dairy in determining sleep relies largely on the role of key nutrients in promoting endogenous melatonin production, milk alternatives would need to have comparable nutrient profiles with those of dairy to support a biologic plausibility to enhance sleep. Although it is certainly worthy of investigation to determine whether dairy products and dairy alternatives differentially affect sleep relative to a control, we are not aware of any study of the association or effect of plant-based beverage consumption on sleep. Food/beverage-specific studies are clearly needed given the complexity of whole food matrices.

In addition to limitations and calls for action highlighted earlier, questions remain about a potential effect of timing of intake of dairy products on sleep. One study indicated that timing of dairy intake may influence its effects on sleep [79]. In that study, light exposure modulated effects of Trp on nighttime melatonin secretion: consuming a Trp-rich breakfast during bright-light daytime conditions led to greater overnight melatonin levels than did consuming the same breakfast under dim-light conditions. It makes sense that timing of intake could affect changes in sleep in response to dairy protein intake because absence of sunlight is the primary catalyst for melatonin production and secretion from the pineal gland. As more data on the effects of dairy and dairy proteins on sleep become available, the effect of timing of intake will be an important factor to consider to ensure that recommendations maximize melatonin synthesis and, consequently, sleep health benefits.

In conclusion, existing observational and experimental data are limited, yet tentatively support a positive relation between dairy products consumption and sleep quality. This is likely achieved through 2 key pathways, implicating increased Trp availability for conversion to melatonin and improvements in the gut microbiome. Although rigorous clinical trials are needed, results of current trials supplementing the diet with either dairy proteins, micronutrient cofactors in melatonin synthesis, or fermented dairy products rather consistently note favorable effects on sleep. The studies appraised in this review provide preliminary evidence on which to build the research base needed to make recommendations targeting dairy product consumption to promote sleep quality.

## Author disclosures

FMZ is a Berrie Fellow in Diabetes Research. The Funding agencies had no role in the conceptualization, design, reporting, or writing of this manuscript. The authors report no conflict of interest to disclose.
